# Preclinical models of vertebral osteomyelitis and associated infections: Current models and recommendations for study design

**DOI:** 10.1002/jsp2.1142

**Published:** 2021-03-02

**Authors:** Kieran Joyce, Daisuke Sakai, Abhay Pandit

**Affiliations:** ^1^ CÚRAM SFI Research Centre for Medical Devices National University of Ireland Galway Ireland; ^2^ School of Medicine National University of Ireland Galway Ireland; ^3^ Department of Orthopaedic Surgery Tokai University School of Medicine Isehara Japan

**Keywords:** animal models, in vivo, infection, spondylodiscitis, vertebral osteomyelitis

## Abstract

Spine‐related infections, such as vertebral osteomyelitis, discitis, or spondylitis, are rare diseases that mostly affect adults, and are usually of hematogenous origin. The incidence of this condition has gradually risen in recent years because of increases in spine‐related surgery and hospital‐acquired infections, an aging population, and intravenous (IV) drug use. Spine infections are most commonly caused by *Staphylococcus aureus*, while other systemic infections such as tuberculosis and brucellosis can also cause spondylitis. Various animal models of vertebral osteomyelitis and associated infections have been investigated in mouse, rat, chicken, rabbit, dog, and sheep models by hematogenous and direct inoculation in surgery, each with their strengths and limitations. This review is the first of its kind to concisely analyze the various existing animal models used to reproduce clinically relevant models of infection. Spine‐related infection models must address the unique anatomy of the spine, the avascular nature of its structures and tissues and the consequences of tissue destruction such as spinal cord compression. Further investigation is necessary to elucidate the specific mechanisms of host‐microbe response to inform antimicrobial therapy and administration techniques in a technically demanding body cavity. Small‐animal models are not suitable for large instrumentation, and difficult IV access thwarts antibiotic administration. In contrast, large‐animal models can be implanted with clinically relevant instrumentation and are resilient to repeat procedures to study postoperative infection. A canine model of infection offers a unique opportunity to design and investigate antimicrobial treatments through recruitment a rich population of canine patients, presenting with a natural disease that is suitable for randomized trials.

## Graphical abstract

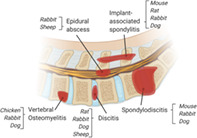

## 1 INTRODUCTION

Vertebral osteomyelitis, also called spondylodiscitis or spondylitis, is a rare disease that mostly affects adults, and is usually of hematogenous origin. Vertebral osteomyelitis is an infection of the vertebral bodies which may involve the adjacent intervertebral disc (IVD) resulting in an associated discitis. Vertebral osteomyelitis, accounting for about 5% of all osteomyelitis cases, has an incidence of approximately 2.4 cases per 100 000 population, and incidence increases from 1.7 per 100 000 in <59 years of age to 25.1 per 100 000 in >80 years of age.^1^ Risk factors for developing an infection include intravenous (IV) drug use, bacterial endocarditis, intervertebral disc degeneration, previous spinal surgery, diabetes mellitus, corticosteroid therapy, or other immunocompromising conditions.^2^, ^3^, ^4^, ^5^, ^6^

Vertebral osteomyelitis most commonly occurs due to hematologically derived seeding, direct inoculation in spinal surgery, or from the invasion of infection from surrounding tissue.^7^ The infection is commonly due to bacteria, but fungi and parasites have also been identified as causative agents. The most commonly implicated organism in spine‐related infections is *Staphylococcus aureus* (methicillin resistance becoming more prevalent), followed by *Escherichia coli*.^8^ Osteomyelitis due to direct inoculation during spinal surgery, particularly after instrumentation, is most often caused by *S. aureus* and *Propionibacterium acnes*, a typically normal inhabitant of skin flora.^7^, ^8^ However, low‐virulence microorganisms such as coagulase‐negative *S. aureus* may induce hematogenous vertebral osteomyelitis, especially in the setting of a prolonged implant‐associated bacteremia.^9^ Most patients that develop hematogenous vertebral osteomyelitis have ongoing co‐morbidities, such as diabetes mellitus, coronary artery disease, immunosuppression, cancer, or renal failure requiring hemodialysis.^10^, ^11^, ^12^, ^13^

Spinal infections have a variable presentation, and as such, vertebral osteomyelitis can be complicated by paravertebral, epidural, or psoas abscesses by direct seeding.^7^ In a study reporting on the complicated presentation of vertebral osteomyelitis, an epidural abscess was reported in 17% of cases, paravertebral abscess in 26%, and intradiscal abscess in 5%.^7^ Motor weakness or paralysis develops in approximately a quarter of patients, with an increased incidence of neuropathy occurring in patients with osteomyelitis of the cervical spine. Overall, neurological complications are common in vertebral osteomyelitis, where 38% of patients will develop neurological symptoms.^14^

### 1.1 Hematogenous dissemination

Hematogenous dissemination is the most common cause of vertebral osteomyelitis.^15^ Adult vertebral bone is highly vascularized with slow high‐volume blood flow via the posterior spinal artery, making it susceptible to bacterial seeding.^16^ Many patients with hematogenous pyogenic vertebral osteomyelitis are predisposed due to underlying conditions such as diabetes mellitus, heart disease and immunocompromising disorders.^8^, ^13^, ^17^ The lumbar vertebral bodies are most often implicated, followed by thoracic and, less commonly, cervical vertebrae, while hematogenous sacral osteomyelitis is rare. Noncontiguous epidural abscesses occur in approximately 10% of the cases that are complicated by abscess.^18^

### 1.2 Direct inoculation

Direct bacterial inoculation in spinal surgery and subsequent postoperative infection is a devastating complication, associated with increased morbidity and/or mortality. In vertebral osteomyelitis, management is further complicated by the avoidance of instrumentation explantation, which would destabilize the spine with potential neurologic compromise. Patients that develop vertebral osteomyelitis require prolonged hospitalization, repeat surgeries for removal of instrumentation and/or debridement, and a long course of IV antibiotics, followed by oral antibiotics. Postoperative infections incur a heavy burden on healthcare systems estimated at one million excess inpatient days and 2.72 billion USD additional costs per year in the US alone.^19^ Approximately 1% of the patients undergoing elective spine surgery without instrumentation are complicated by postoperative infection and incidence increases when the hardware is used, despite stringent aseptic surgical technique and prophylactic antibiotic protocols.^20^ Implant‐associated infection is complicated by biofilm formation, where bacteria readily adhere to implant surfaces, developing a biofilm layer over several days, reducing antibiotic susceptibility by 100 to 1000 times.^21^

### 1.3 Extension of primary infection site

Primary vertebral osteomyelitis can be complicated by an extension of the initial infection. Infection may extend posteriorly as an epidural abscess, subdural abscess, or even meningitis, and are more often associated with gram‐positive bacterial infection than gram‐negative bacterial infection.^16^, ^22^, ^23^ Anterior or lateral extension of infection can lead to paravertebral, mediastinal, retroperitoneal, or psoas abscess.^3^ Infection can occur in spinal elements other than the vertebral bodies, including the posterior spinous processes, the facet joints, and the pedicles.^24^ Thoracic vertebral infections have even been recorded to extend into the pleural space to produce an empyema.^25^

### 1.4 Diagnosis

The diagnosis of vertebral osteomyelitis can be challenging, as infection may be insidious, often resulting in delayed identification of the condition and infecting organism. When clinical suspicion warrants investigation, diagnosis can be confirmed with the use of magnetic resonance (MR) imaging, microbiological cultures, and tissue biopsy examinations. Vertebral osteomyelitis is identified by high signal intensity on T2 weighted MR images.^26^ Molecular diagnostics are not routinely used when investigating vertebral osteomyelitis; however, negative bacterial cultures spur the use of a panel polymerase‐chain‐reaction (PCR) analysis to identify microbial DNA in biopsies.^27^ This enables the detection of less common microorganisms, such as anaerobic bacteria, *Brucella* and *Bartonella* species.^27^ Broad‐range PCR is limited by reduced sensitivity and specificity, which dramatically decrease due to the probability of contamination and cannot provide an antibiotic resistance profile for the microorganisms.^27^

### 1.5 Clinical management

At present, there are no data from clinical trials to inform specific antimicrobial regimens for vertebral osteomyelitis and associated infections, nor are there guidelines on the duration of antibiotic therapy. The choice and duration of therapy cited in case reports may be associated with the extent of infection or with patient‐specific considerations, offering little value to prescribing guidelines. Given the increasing incidence of spine‐related infections and the significant morbidity and mortality of this condition, further, preclinical research and clinical trials are needed to elucidate the variable onset and progression of this debilitating complication.^28^ It is challenging to design and undertake clinical trials for this complicated disease process, considering the low incidence and high heterogeneity of induction and presentation of infection with various implicated organisms.

This paper provides an overview of the characterized preclinical models of vertebral osteomyelitis, highlighting the strengths and weaknesses of each model and suitability for controlled trials of treatment strategies. Since the first documented model of vertebral osteomyelitis was developed in chickens in 1971 by Wise *et al*, significant work progress has been made in developing a standardized model to replicate human disease as models for antimicrobial therapies and surgical management of vertebral osteomyelitis.^29^ In the present review, the authors critically analyze characterized spine‐related infections in each relevant animal model to evaluate reproducibility, clinical relevance and representation of natural disease. The authors also make a case for the use of veterinary patients (specifically dogs) presenting to clinic with natural disease as a suitable cohort for animal trials to test the efficacy of antibiotics and surgical treatments.

## 2 MODELS OF SPINAL INFECTIONS

**FIGURE 1:**
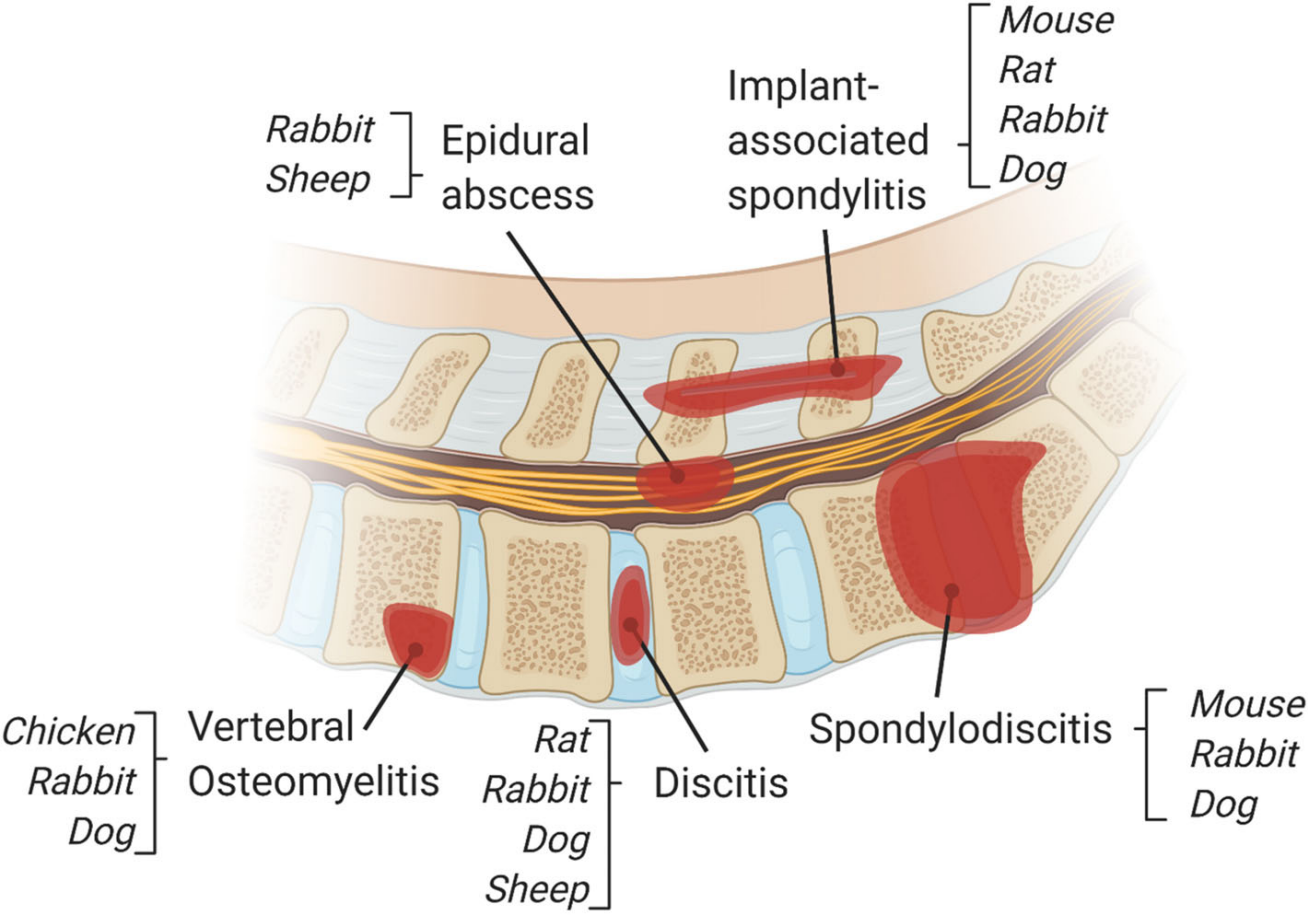
Models of induced vertebral osteomyelitis and associated spine‐related infections and validated species. Definitions of each infection; vertebral osteomyelitis is an infection of the vertebral body, discitis is a localized infection of the IVD, spondylodiscitis is an infection of the IVD with adjacent vertebral involvement, implant‐associated spondylitis includes implantation of an inoculated foreign body, epidural abscesses are extensions of localized infection or associated with disseminated infection. Models characterizing epidural abscess are associated with vertebral osteomyelitis or disseminated infection

While the human disease is referred to as vertebral osteomyelitis (infection of the vertebral bone) or discitis (infection of the IVD) or spondylodiscitis (a combination of both), animal models replicating these conditions use highly variable language and definitions surrounding the type of infection (Figure 1). Thus, the authors have attempted to discuss these models as uniformly as possible for comparability. To summarize the additional terminology used below; implant‐associated spondylitis includes implantation of an inoculated foreign body, disseminated infection describes the spread of an infective organism throughout the body, abscesses are complications of localized disease and/or disseminated infection, and acute pyogenic spondylodiscitis is a subset of spondylodiscitis with the production of pus.

Various animal models of vertebral osteomyelitis and associated infections have been investigated in recent decades. Mouse, rat, chicken, rabbit, dog, and sheep models are summarized in Table 1. Models have been sub‐classified for ease of summation. Small‐animal models, such as mouse and rat, and large‐animal models, including dogs and sheep, have been discussed to evaluate the method of infection induction used, the following characteristics of infection that were generated and investigations performed to assess the response to infection. Rabbits have been most extensively studied and thus have been discussed separately. Case‐reports of veterinary studies have also been included to highlight the subset of studies that investigate naturally occurring disease in animals.

**TABLE 1:** Animal models of vertebral osteomyelitis and associated infections

Species	Model of infection	*Bacterial inoculum*, and dose	Method of bacterial introduction	Investigation/detection strategies	Reported findings	Ref.
Chicken	Vertebral osteomyelitis	Coagulase negative, nonhemolytic *S. albus*/*S. aureus*, 1.7 × 10^8^ CFU	Disseminated—IV injection	Microbiology, histological evaluation	*Macro*: Active infections observed with disrupted vertebrae and spinal cord compression. *Histo*: Abnormalities in T5‐7 regions of the vertebral column, seen as clefts in the growth plates.	29
Mouse	HLA‐B27 transgenic, Disseminated infection	*Yersinia enterocolitica*, 10^2^‐10^9^ CFU, 0.1 mL	Disseminated—Intraperitoneal infection	Histological evaluation, spinal motility, X‐ray, bacterial cultures.	*Clin*: Hind limb paralysis progression with advanced infection. Altered lumbar spine but no evidence of arthritis. *Macro*: All animals that developed paralysis were found to have abscesses within the spinal column along the length of the spine. *Histo*: Inflammatory cell infiltration and vertebrae destruction were observed.	30
	IRF‐1^−/–^knockout, disseminated brucellosis	*Brucella melitensis* 16M (ATCC23456), 1 × 10^7^ CFU	Disseminated—Intraperitoneal injection	Bioluminescent imaging (EZ::TN/*lx* probe)	Bacterial signal localized in the tail region of the spine during the later stage of systemic infection. *Histo*: Indicative of chronic infection in the osteoarticular tissue of the tail.	31
	Implant associated —Vertebral osteomyelitis	*S. aureus* (Xen36) 10^2^‐10^4^ CFU, 2 mL	Implant associated—Inoculated stainless steel implant into L4 spinous process	Bioluminescent imaging, quantitative bacterial cultures, neutrophil recruitment.	*Clin*: Inoculum of 10^3^ CFU was sufficient to establish a chronic implant infection while avoiding wound breakdown. *Histo*: Neutrophil fluorescence peaked after 3 days, while the infected group demonstrated continued inflammatory infiltration through to postoperative day 35.	32
Rat	Implant associated—Vertebral osteomyelitis	*S. aureus*, 10^2^‐10^6^ CFU, 0.01 mL	Implant associated—Titanium screw implantation at T10/L1 after laminar decortication	Bacterial cultures, biofilm analysis, histological evaluation.	*Clin*: Active infection was established in all inoculated groups. Bacteria were detected around screws at inoculum site. *Macro*: Defects were identified by tissue loss and replacement with granulomatous tissue. *Histo*: Suppurative inflammation was present in the group that received 10^6^ CFU/0.01 mL.	33
	Discitis	*S. aureus*, 10^2^‐10^6^ CFU, 0.1 mL	Direct inoculation—Injection into the intervertebral tail segment	In vivo monitoring, histological evaluation, postmortem imaging.	*Macro*: IVD space reduction and evidence of osteophyte formation on radiographic analysis. *Histo*: Neutrophil infiltration, destruction of the IVD tissue and vertebral endplates.	34
	External fixation bacterial colonization	None, Pin exposed to the external environment.	Implant associated—Titanium pin insertion in C3, C4 and C5	Quantitative bacterial cultures.	*Bact*: Pins underwent bacterial colonization. Nitric oxide releasing pins were less colonized than nontreated pins. *Histo*: No active infection recorded	35
Rabbit	Vertebral osteomyelitis	*S. aureus*, Newman strain (NTCC 8178), 5‐15 × 10^3^ CFU, 0.2 mL	Direct inoculation—Percutaneous injection into L5‐6 IVD	MRI, ultra small superparamagnetic iron oxide (USPIO) particles to localize macrophages, histopathological evaluation.	*MRI*: Signal intensity increased in the endplates of all animals in the infection and sterile‐inflammation groups. In the infection group, a signal increase from USPIO was more significant than the sterile‐inflammation group, showing that USPIO can discriminate infectious and noninfectious inflammation.	37
		*S. aureus*, Newman strain (NTCC 8178), 5‐15 × 10^3^ CFU/mL, 0.2 mL	Direct inoculation—Percutaneous injection into L3‐4 and L5‐6 IVD	MRI, ultra small superparamagnetic iron oxide (USPIO) particles to localize macrophages, histopathological evaluation.	*MRI*: One day after USPIO particles were administered, T1‐weighted images demonstrated increased signal intensity in all infected vertebrae. *Histo*: Replacement of healthy bone marrow of vertebrae by an inflammatory infiltrate on hematoxylin‐eosin staining.	38
	Brucellar spondylodiscitis	*Brucella melitensis*, 3 × 10^8^ CFU/mL, 0.1 mL	Implant associated—Kirschner wire insertion into L6 and bacterial inoculation on a gelatin sponge	MRI, macroscopy, histological evaluation.	*MRI*: Spondylodiscitis, characterized by T2 hyper‐intensity and endemic inflammation in two or more structures. *Histo*: Infiltration of inflammatory cells on histology, predominantly lymphocytes and monocytes.	39
	Discitis	*S. aureus*, 10^6^ CFU, 0.1 mL	Direct inoculation—Inoculation into intradiscal space of lumbar spine	Bacterial cultures.	*Bact*: Bacterial growth observed in both controls and antibiotic treated groups.	40
		*S. aureus*, 2 × 10^4^ CFU/mL, 0.05 mL	Direct inoculation—Inoculation into intradiscal space of L4/5	MRI, histological evaluation.	*MRI*: Discitis was induced at all inoculation sites, heterogeneous intensity of T1‐weighted images of the IVD and hyper‐intensity of T2‐weighted images of the bone marrow of the vertebra. *Histo*: The IVD presented with the destruction of the NP with inflammatory and fibroblastic cell infiltrates.	41
		*S. aureus*, 10^1^‐10^3^ CFU	Direct inoculation—27.5 gauge needle insertion into lumbar IVD and inoculation	Bacterial cultures.	*Bact*: Colony growth from untreated discs, indicating infection in all inoculated groups. Cefazolin and vancomycin pretreated discs did not grow bacterial colonies.	42
	Vertebral osteomyelitis—Spinal epidural abscess	*S. aureus*, 10^8^ CFU, 0.01 mL	Direct inoculation—Laminectomy and inoculum in epidural space in thoracolumbar junction	Neurological evaluation, bacteriology, biochemistry.	*Clin*: Progressive neurological deficits were observed in 90% of animals, characterized by lower extremity weakness, sphincter dysfunction, and abnormalities of gait. *Macro*: Spinal abscess confirmed in 95% of cases.	43
	Implant associated—Vertebral osteomyelitis	Methicillin resistant *S. aureus*, 10^2^‐10^3^ CFU	Implant associated—Partial laminectomy and wire implantation (inoculated with MRSA) at T13, L3 and L6	Bacteriology, organ biopsies, blood cultures.	Postmortem quantification of bacteria showed extremely high bacterial burdens at inoculum sites. 10^3^ CFU induced infection consistently. *Bact*: MRSA inoculation consistently established a device‐centered infection after 7 days in this model.	44
		Methicillin resistant *S. aureus*, and *E. coli* 10^3^ CFU	Implant associated—Partial laminectomy and wire implantation (inoculated with MRSA) at T13, L3 and L6	Radionuclide imaging, histological evaluation.	*Histo*: confirmed the onset of infection in each animal. *Imag*: Gram‐positive infections could be observed on nucleotide imaging by ^111^In‐WBC accumulation while gram‐negative had little accumulation of ^111^In‐WBC.	45
		*S. aureus*, 10^3^ CFU, 0.05 mL	Implant associated—L4/5, spinous process removed, decortication of lamina, 26—gauge surgical wire implanted	Aerobic swab, bacterial cultures.	*Bact*: In nonantibiotic treated group, the swab and tissue cultures grew *S. aureus* in all five rabbits. Rabbits that received instrumentation and cefazolin before surgery did not grow *S. aureus*.	46>
Sheep	Discitis	*S. epidermidis*, 2 × 10^1^ CFU, 0.1 mL	Direct inoculation—27.5 gauge Intradiscal inoculation	Macroscopy, histological evaluation	*Histo*: Central discitis with the destruction of endplates and extensive hemorrhage. Prophylactic antibiotics prevented infection *Clin*: Antibiotics could not resolve established infection.	53
		*S. aureus*, 10^3^ CFU, 0.1 mL	Direct inoculation—Induced disc degeneration and discography with inoculation	Radiography, histological evaluation	*Macro*: Discitis detected in nonantibiotic treated sheep. *Macro*: Evidence of endplate erosion and disc thinning which progressed over 12 weeks and abscess formation. *Histo*: Extensive inflammatory response.	55
Dog	Vertebral osteomyelitis	*S. aureus*, *Pseudomonas* and *E. coli*	Direct inoculation—Fluoroscopic intradiscal inoculation	Radiography, histological evaluation	*Histo*: Acute inflammation, fusion of the adjacent vertebrae became apparent in 8 weeks. *Clin*: The degree of the disease process was more advanced in the *S. aureus* group and less severe in the *Pseudomonas* group.	57
	Discitis	*S. aureus*	Direct inoculation—Inoculation of vertebral body using gauze	Radiography, histological evaluation	*Histo*: Acute inflammation started within 1 or 2 weeks and subsided by 5 or 6 weeks. *Macro*: In 55% of the dogs, the inflammation was confined within the vertebral body, in 10% it invaded into the IVD, and in 35% inflammation invaded into the anterior longitudinal ligament.	58
	Pyogenic spondylodiscitis	*S. aureus* (29 213; ATCC), 10^1^‐10^5^ CFU/mL, 0.2 mL	Direct inoculation—Partial discectomy (T12‐L1) and induced end plate damage	Histological evaluation, quantitative bacterial cultures.	*Bact*: Inoculum concentration was optimized at 10^2^ CFU to reliably induce infection. *Histo*: Established infection characterized by inflammatory cell infiltration and osteonecrosis was observed with the spreading of infection to the adjacent vertebra.	59
		*S. aureus* (29 213; ATCC), 10^8^ CFU/mL, 0.1 mL	Direct inoculation—Partial discectomy (L2‐L3) and induced end plate damage	MRI, bacterial detection by PCR	*MRI*: Confirmed the presence of infection in all animals through observation of characteristic changes in signal intensity on T1‐ and hyper‐intensity on T2‐weighted images in the infected IVD and adjacent vertebrae.	60

Abbreviations: Bact, bacteriology; Clin, clinical presentation; Histo, histological evaluation; Imag, radionucleotide imaging; Macro, macroscopic findings; MRI, magnetic resonance imaging report.

### 2.1 Rodent models

Mouse models of vertebral osteomyelitis have been investigated in both hematogenous and direct inoculation models of infection have been studied in mice. *Brucella melitensis* (1 × 10^7^ Colony forming units—CFU) and *Yersinia enterocolitica* (10^2^‐10^9^ CFU) have been injected via intraperitoneal inoculation in Interferon regulatory factor knockout (IRF−/−) and Human leukocyte antigen B27 transgenic (HLA‐B27) mice, respectively.^30^, ^31^ These studies characterized the subsequent infection using histological evaluation, spinal motility assessment, bacterial cultures and bioluminescent imaging, albeit variably with no standardization across studies. Clinically, hind‐limb paralysis was described in HLA‐B27 mice.^30^ All animals that developed paralysis were found to have abscesses within or to compress the spinal column along the length of the spine and histology revealed inflammatory cell infiltration and vertebrae destruction. Bioluminescent imaging of IRF−/− mice was used to localize ongoing infection in the osteoarticular tissue of the mouse.^31^ In addition to these disseminated models of infection in mice, an implant‐associated model was developed using inoculated stainless steel implants into the spinous process.^32^

Several rat models of infection have included lumbar vertebral osteomyelitis, implant‐associated infection, isolated discitis and external fixation colonization characterization.^33^, ^34^, ^35^10^2^‐10^6^ CFU of *S. aureus* was used as an inoculating agent in infectious models.^33^, ^34^, ^36^ Titanium screws inoculated with *S. aureus* have been used to reproduce an implant associated infection to investigate the efficacy of antibiotic therapies.^33^, ^36^ In the study of pin tract bacterial migration, skin flora colonized the titanium pins in the external fixation model.^35^ These studies used *in vivo* monitoring of bioluminescent bacteria, histological evaluation, bacterial cultures, biofilm analysis and postmortem radiographic imaging. In the model of isolated discitis, radiographic analysis revealed reduced IVD height, evidence of osteophyte formation and discitis, while destruction of the IVD and vertebral endplates was observed histologically.^34^ A significant osseous infection was confirmed in implant‐inoculated model with localized tissue destruction and loss of bone; soft tissue was filled with young granulation tissue characterized by infiltration of inflammatory cells. On the other hand, the colonization model of external fixation with skin flora did not lead to active infection in the presented study, making it unsuitable for the study of spine infection however, this model is useful to investigate material coatings to inhibit bacterial colonization of implants and subsequent infection. These rat models replicate direct inoculation and implant‐associated infection, which may be useful to study antimicrobial coatings, biofilm formation and resistance to antibiotics. Small‐animal models are useful specific studies that require rapid maturity of off‐spring, including genetically‐modified strains and gene‐knockout animals. These animals are easily housed in preclinical facilities and are relatively inexpensive compared to larger animals. Mouse models are highly suited to the study of disseminated infection with spinal complications and hematogenous induced infection, albeit more challenging for delivery of therapeutic agents and antimicrobials while rat models are suitable for the study of spinal‐instrumentation.

### 2.2 Rabbit models

To date, rabbit models of spine infections have been most intensely investigated over any other animal models. Studies have explicitly included models of lumbar vertebral osteomyelitis,^37^, ^38^ brucellar spondylodiscitis,^39^ isolated discitis,^40^, ^41^, ^42^ vertebral osteomyelitis with complicated abscess,^43^ and implant‐associated spine infection.^44^, ^45^, ^46^ Bierry et al investigated a model of vertebral osteomyelitis using *S. aureus* (Newman strain, NTCC 8178, 5‐15 × 10^3^ CFU).^37^, ^38^ The authors used ultra‐small superparamagnetic iron oxide (USPIO) nanoparticles to localize macrophages. They determined that magnetic resonance imaging (MRI) signal intensity combined with USPIO signal could discriminate between infectious osteomyelitis and noninfectious inflammation, as confirmed by histology. This may be a useful indicator in diagnostic investigations with queried inflammation on MRI.

In 1998, Guiboux et al described the first implant‐associated spine infection model in rabbits.^46^ Since it is of particular interest to produce infection associated with, and complicated by, in situ instrumentation, a previous model of discitis was combined with an instrumentation technique to create a postoperative instrumentation‐associated infection model.^46^ In the nonantibiotic treated group, the swab and tissue cultures grew *S. aureus* in all five rabbits. Rabbits that received instrumentation and first‐generation cephalosporin, cefazolin before surgery did not grow *S. aureus*. This study showed that prophylactic antibiotics can effectively inhibit infection after direct inoculation during surgery. Models of vertebral osteomyelitis associated with spinal instrumentation have been developed using Methicillin resistant *S. aureus* (MRSA) (10^2^‐10^3^ CFU) by partial laminectomy, and wire implantation (inoculated with MRSA) at several noncontiguous vertebral levels in the lumbar region.^44^, ^47^, ^48^, ^49^ Postmortem quantification of bacteria showed extremely high bacterial burdens at inoculum sites where the inoculated wire had been placed. MRSA showed a consistent capability to establish an instrument‐associated infection after 7 days in this model. ^111^In‐labeled (Indium‐111 radioactive isotope) white blood cell (WBC) imaging and histological studies confirmed the induction of infection in each animal. Gram‐positive infections were observed on nucleotide imaging by ^111^In‐WBC accumulation while gram‐negative had little accumulation of ^111^In‐WBC. A rabbit model of vertebral osteomyelitis has been complicated by epidural abscess formation through laminectomy and *S. aureus* (10^8^ CFU) inoculation.^43^ Progressive neurological deficits were observed in 90% of animals, characterized by lower extremity weakness, sphincter dysfunction, and abnormalities of gait. Spinal abscess confirmed in 95% of cases.^43^ This model presents a challenging infection for antibiotic therapies, considering the reduced antibiotic penetrance into abscesses.^50^

Infectious discitis has been induced by intradiscal inoculation using *S. aureus* (10^4^‐10^6^ CFU).^40^, ^41^ Discitis was confirmed in the inoculated levels by different intensity on T1‐weighted images of the IVD and hyper‐intensity of the adjacent vertebrae on T2‐weighted images.^41^ The infected IVD presented with the destruction of nucleus pulposus tissue and necrosis with associated inflammatory and fibroblastic cell infiltration.^41^ A further study found that while vancomycin reduced the overall bioburden within a contaminated surgical site of posterolateral fusion, the addition of the vancomycin to the demineralized bone matrix reduced the fusion capability of the demineralized bone graft.^51^ Fusion rates were restored however with an ileal crest graft.

Spondylodiscitis has been induced by a *Brucella melitensis* (3 × 10^8^ CFU) inoculated gelatin sponge, co‐implanted with a Kirschner wire (K‐wire) insertion into L6.^39^ Spondylodiscitis was observed on MRI, characterized by T2 hyper intensity, regional inflammation involving the vertebra and diffuse marrow edema with paraspinal abscess. Infiltration of inflammatory cells was observed on histology, predominantly consisting of lymphocytes and monocytes. Rabbit models of infection have been developed to replicate hematogenous seeding, direct inoculation and instrumentation‐associated infection. Despite their popularity due to sufficient vertebral size to sustain instrumentation and ease of handle ability, questions remain over their susceptibility to infection and are limited by their intolerance to repeat procedures.

### 2.3 Ovine models

Large‐animal models have advantages over small‐animal models in the study of spine disease as they have more relevant anatomy, allow for easy IV access for antibiotic administration and support clinically relevant instrumentation.^52^ Fraser et al described the first animal model of discitis by direct inoculation into the sheep IVD to investigate the efficacy of IV antibiotics in 1989.^53^, ^54^ This study demonstrated the value of prophylactic antibiotics in effectively preventing infectious discitis; however, antibiotic treatment was not sufficient to eliminate an established *S. epidermidis* infection, highlighting the limitations of antibiotic therapies alone. A further ovine model of discitis, using direct intradiscal inoculation by *S. aureus* describes similar resulting infection.^55^, ^56^ Discitis was detected in nonantibiotic treated sheep, characterized by endplate erosion and disc thinning on gross examination and extensive inflammation on histological evaluation.^55^

### 2.4 Canine models

Canine models of spine infection were first described in 1991 in Japan, induced by *S. aureus*, *Pseudomonas*, and *E. coli* intradiscal inoculation.^57^ Results demonstrated a more advanced disease with increased tissue destruction in *S. aureus* infection.^57^ More sophisticated models of spine infection have since been developed to produce a complicated acute pyogenic spondylodiscitis in canine models.^58^ Chen et al described a model of partial discectomy and endplate damage with direct inoculation of *S. aureus* in the lumbar spine.^59^ Inoculating concentrations greater than 10^2^ CFU resulted in higher mortality or were complicated by surgical wound dehiscence. In contrast, lower bacterial concentrations did not reliably induce infection. 10^2^ CFU, the optimized inoculating concentration, induced an inflammatory process characterized by inflammatory infiltration and osteonecrosis with spreading of infection to the adjacent vertebra.^59^ This model was further investigated by MRI examination of the spinal infection, where characteristic changes of a heterogeneous signal on T1‐ and hyper‐intensity T2‐weighted images confirmed active infection in the involved IVD and adjacent vertebrae.^60^ Canine and ovine models are optimal for studies investigating instrumentation associated infection considering the facilitated use of relevant medical devices available on the market, and the surgical management of infection and neurological complications such as spinal instability and, spinal cord compression given the suitable anatomy for repair which would be far more technically challenging in smaller models.

### 2.5 Case‐reports of veterinary patients with natural disease

Veterinary reports may be useful to understand common causative organisms in animals and natural disease presentation and progression. Several case reports exist of spinal infections described in animals. These reports may be useful to determine the fidelity of artificial models to environmentally induced disease. Vertebral osteomyelitis, complicated and uncomplicated, has been well described in dogs.^61^, ^62^ Vertebral osteomyelitis in dogs is most commonly caused by *Staphylococus pseudintermedius*.^61^ while bacteria including *Brucella canis*, *Streptococcus* spp., *Escherichia coli* have also been frequently cultured from infected tissues. Reports of infections caused by *Salmonella*, *Bacteroides* spp., *Bordetella* spp., *Pasteurella multocida*, and *Proteus* spp. have also been recorded.^62^, ^63^, ^64^, ^65^, ^66^ Fungal species were also identified in discospondylitis in dogs, such as *Aspergillus terreus*.^67^ *Scedosporium apiospermum* infection, an eutrophic filamentous fungus, has been recorded in a canine case report of osteomyelitis and discospondylitis.^68^ No randomized clinical trials have investigated antibiotic regimens in dog patients, and clinical management has mostly followed practices similar to human case reports.^69^, ^70^, ^71^, ^72^ Nonhuman primates have also been studied, as posterior paralysis and spinal osteomyelitis have been described in a case report of a Rhesus monkey with *Coccidioides* spp. infection.^73^ Radiography revealed soft tissue swelling and bone lysis in the thoracic spine of a monkey with low limb paralysis. On postmortem, an epidural empyema was found in the area of the dorsal spinous process of the 11th thoracic vertebra and extending around the spinal cord, positive for *Coccidioides* spp.^73^ These veterinary reports are a valuable resource, validating animal models against natural disease in animals and comparing natural disease in humans and animals. The use of veterinary patients in trials of antibiotic therapies would be largely beneficial and more relevant than preclinical models of induced infection given the presence of underlying comorbidity and susceptibility these animals have towards disease, making them more representative of human disease.

## 3 LIMITATIONS OF CURRENT MODELS

The animal models described in Table 1 have been optimized to be reproducible when sufficient CFU count has been used in inoculation. Early studies by Guiboux et al and Fraser et al described rabbit and sheep spinal infection models and highlighted the efficacy of prophylactic antibiotics.^42^, ^53^ Results from these studies are inconsistent with reports from clinical studies as some patients will develop postoperative infection despite the administration of prophylactic antibiotics. These findings are of course confounded by the increased susceptibility some clinical patients will have to postoperative infection such as primary immunodeficiency, underlying comorbidities such as diabetes and other risk factors as discussed previously. These models may be further optimized to include longer time points, and higher inoculation doses to readily detect subsequent infection comparable to clinical patients.

Models of vertebral osteomyelitis associated with spinal implants are of particular importance given the prevalence of this challenging complication post instrumentation. A robust model that promotes biofilm formation with relevant causative microorganisms is essential to trial new antimicrobial therapies and implant surface treatments. Ofluoglu et al successfully mimicked pedicle screw implantation in the rat spine; the most commonly implanted hardware in spine procedures.^33^ Tissue and implant cultures were performed to detect signs of osteomyelitis and confirmed using histological evaluation. Contrary to other models that cite 10^3^ CFU as a sufficient inoculation strategy, this model required 10^6^ CFU concentration to induce bone destruction, and inflammatory cell infiltration.^33^ Several rabbit models of instrumentation‐associated infection have also been replicated using Kirschner‐wires.^44^, ^45^, ^46^ In this model, Poelstra et al studied several sites of infection in noncontiguous levels in the same animal.^44^ This established an internal control in all animals allowing for effective cross‐comparison of treatment regimens and implanted materials to monitor the development of biofilm in implant‐associated infections.^44^ Such a model is confounded by the systemic immune response induced by a localized infection. The surgical approach used in this model involved a partial laminectomy that created a “dead space” allowing inoculated bacteria to thrive, resembling postoperative infections in the clinic. Further limitations are discussed below to consider animal species, inoculating bacteria, route of inoculation and methods of evaluation.

### 3.1 Animal species

Rabbit models of osteomyelitis and spondylitis have been popular due to their predisposition to infection over other animals; however, this susceptibility should be considered for cross species comparison. Rabbits have large enough vertebrae for models that use instrumentation to investigate biofilm formation and penetrance, while being less costly than dogs. While rats are even less expensive than rabbits, their smaller spines are not suitable large instrumentation and difficult IV access impedes antibiotic administration. Furthermore, animal models smaller than rabbits make it challenging to investigate complications such as neurological compromise due to abscess or vertebral instability because of their small spines. Mouse and chicken models may be good candidates when investigating hematogenous osteomyelitis as vertebral complications have been characterized .^29^, ^30^, ^31^ Mice can be genetically modified to study immunomodulation and its role in developing an infection. A relevant, validated mouse model would be of great benefit in this field, to reliably investigate potential therapies and new devices in a preclinical setting. Promising therapies may progress to testing in larger animal models for further validation and eventually in clinical trials. More often, canine patients are presenting to veterinary clinics with complicated disease and compounding morbidity.^74^ Canine patients receive chemotherapy and undergo complex surgeries.^74^ Often, aged animals are subject to similar medical interventions as humans. These canine patients are highly valuable as they replicate human‐like disease. While presenting data of high clinical value, a study involving these veterinary patients would incur high‐costs with logistical and ethical challenges.

### 3.2 Bacterial species and sufficient inoculum

Current animal models of spinal infections have primarily focused on postoperative infection with *S. aureus* as the inoculating microorganism, given its high incidence in humans. An overview of existing animal models in each given species is outlined in Figure 1. Future models must be tunable to evaluate many causative microorganisms, whether bacterial or fungal given the variety of causative agents listed in numerous case reports. Table 1 lists the microbial species implicated in animal osteomyelitis, where *S. aureus* is the most commonly used bacterial species. Some research groups utilized locally sourced bacterial strains from clinical biopsies, while others used the American Type Culture Collection (ATCC) strains.^59^ While it can be argued that *S. aureus* isolated from patient populations may be clinically relevant, this does not allow for standardization of animal models, and thus a characterized strain such as ATCC classified microorganisms should be promoted.

In animal models of osteomyelitis, specific inoculation quantities have been identified. The number of bacteria needed, dependent on species and bacterial species should be between 10^3^ to 10^8^ CFU per inoculation to establish active infection reliably. However, 10^4^ to 10^5^ CFU should be considered when investigating infection rates when comparing implant designs.^75^, ^76^ In spinal infections models discussed here, inoculation doses range from 2 × 10^1^ to 3 × 10^8^. Standardization of inoculation dosages would be useful for cross‐study comparisons to compare potential novel therapeutic strategies against one another, although conclusions would be speculative. Currently, it is recommended to perform a dose‐response pilot study to determine optimal inoculation concentration to avoid too few infections (low bacterial count) or overt infection leading to complications such as wound dehiscence and systemic sepsis (high bacterial count).

### 3.3 Routes of inoculation

The outlined studies have used various inoculation routes including; intradiscal (percutaneous and open), IV, intragastric, intraperitoneal, and direct inoculation into the surgical field. This also includes immersion of implant or instrumentation in microbial culture or establishing biofilm on implants prior to insertion into the vertebra.^35^, ^39^ Inoculation methods that induce systemic infection with vertebral/spinal involvement aim to replicate hematogenous infection, while inoculation with concurrent instrumentation preferentially aims to reproduce an environment of postoperative infection. These various methods aim to reproduce the spectrum of clinical presentations of infection to highlight potential susceptibility to infection such as zones of hematological stasis that increases the risk of hematogenous seeding and subsequent infection. Implant associated inoculation on the other hand is often reproduced in trials of material coatings to investigate the formation of biofilm and antibiotic penetration. The route of inoculation therefore is largely dependent on the study goals and not necessarily a reflection of the natural disease process.

### 3.4 Methods of evaluation

One of the most considerable limitations of animal models investigated thus far is the heterogeneity and lack of specificity in outcome reporting. While most studies include a form of imaging (X‐ray or MR), histological evaluation and microbiological cultures, the results are reported using nonstandardized grading criteria and quantitative measures while histology is invariably qualitative. Microbiological evaluation is primarily reported as nominal data, confirming the presence or absence of microorganism growth. Initial studies recorded clinical features such as the development of neuropathy and paralysis; however, these findings are not routinely reported.^30^, ^43^ Diagnosis and grading of severity of osteomyelitis in preclinical models must be evaluated by radiographic evaluation, microbiological analysis, and histological grading using standardized criteria. Radiographic criteria for diagnosis and evaluation of the severity of the infection is likely to have been based upon previously described criteria in the context of nonvertebral osteomyelitis.^77^, ^78^ Histological evaluation assessed the bone periosteum, cortex and medullary canal, characterizing the degree of granulation, presence of polymorphonuclear leukocytes, abscess formation and tissue destruction. While negative culture may rule out ongoing infection, a positive culture should be confirmed using PCR to cross‐match against the inoculating strain of bacteria to rule out contamination. More recent studies, have used ultra‐small superparamagnetic iron oxide (USPIO) particles to localize macrophages to discriminate between infectious and noninfectious inflammation by MR imaging increasing the specificity of diagnosis of infection on MR imaging.^38^

## 4 MODEL SUITABILITY FOR INVESTIGATING TREATMENT STRATEGIES

### 4.1 Efficacy of antibiotic therapy

In general, antibiotics should be withheld until the infectious microorganism has been identified, which is most often the case, provided that the patient was not previously treated with antibiotics before culture samples were taken.^16^ No randomized controlled trials have been performed to study the efficacy of antimicrobial therapy in vertebral osteomyelitis, and recommendation online of therapy is mainly derived from observational studies. A retrospective study of 120 participants with clinically diagnosed vertebral osteomyelitis of various microbial origin was treated with appropriate IV regimens for 32 days on average. An infection clearance rate of 91% at six months.^79^ A meta‐analysis investigating antibiotic therapy for the treatment of varying presentations of osteomyelitis produced an average eradication rate was 79% after 1 year across 22 studies.^80^ Differences in antibiotic therapy did not significantly affect the outcomes, except in implant‐associated infection where rifampin was superior.^81^ Controlled trials do not yet suggest the optimal duration of therapy, and antibiotic regimen recommendations range from 4 to 6 weeks,^79^ up to 3 months.^82^ Patients with persistent abscesses and retained instrumentation often require prolonged antibiotic regimens.^81^, ^83^

Studies of antibiotic activity examine many facets of use including prophylaxis, IV administration, localized slow‐release formulations and device coatings. Fraser et al examined the efficacy of IV antibiotics in the prevention and treatment of iatrogenic discitis in an ovine model.^53^ Several studies have investigated various routes of antibiotic administration including, IV administration, local delivery via collagen sponges and antibiotic beads in the surgical site which are all effective at reducing risk of infection however superiority of any dose, drug or route has not been demonstrated.^84^ While prophylactic antibiotics effectively inhibited infection establishment, antibiotic treatment of preexisting infection failed to arrest the progression of discitis.^53^ Similarly, Walters et al failed to abolish established discitis with the antibiotic, cefazolin.^54^, ^55^ Guiboux et al examined the effect of prophylactic antibiotic use in their rabbit model of iatrogenic IVD infections.^42^ In this seminal study, IV cefazolin or vancomycin effectively prevented postoperative discitis in *S. aureus* inoculated IVD.^42^ The same group furthered this study by examining antibiotic efficacy in spinal instrumentation‐associated infection model using inoculated surgical wire implantation around facet joints.^46^ In a model that otherwise produced an established *S. aureus* induced infection, prophylactic antibiotics effectively inhibited infection.^46^ Similarly, a rabbit model of inoculated Kirschner‐wire implantation has been used to study the prophylactic effect of vancomycin powder, and gentamicin loaded poly(lactic‐co‐glycolic acid) (PLGA) spheres delivered into the surgical site before wound closure.^85^, ^86^ Vancomycin treated rabbits returned negative bacterial cultures from surgical site swabs in all cases whereas gentamicin microspheres reduced the incidence of infection from 75% down to 38% in the same rabbits.^85^, ^86^ Vancomycin‐loaded PLGA microspheres obtained similar results, using a lower dose of vancomycin.^87^ These models offer a gold standard, or at least a validated negative control group, to compare new prophylactic strategies. However, no investigations performed thus far have successfully eradicated an established infection.^88^ While these models may be useful to simulate hematogenous or instrumentation‐associated infection, their use for treatments strategies remained to be elucidated. There is a need for suitable validated models to examine antibiotic penetrance and dose depending on means of administration in infections derived from associated bacteria.

### 4.2 Surgical management

Surgical intervention is rarely performed, though it may be appropriate in spine‐related infections, indicated by (a) neurologic deficits. (b) Presence of abscesses in need of drainage. (c) Vertebral collapse and/or spinal instability with or without cord compression. (d) Recurrence of disease despite appropriate antimicrobial therapy.^15^, ^89^ No randomized trials are evaluating surgical management of vertebral osteomyelitis.^15^

It is common practice to administer an additional six‐week “tail” of oral antibiotics in recurrent or chronic infection, although there is little evidence to guide management. When spinal instrumentation is required for stabilization, timely implantation may be safe in the setting of appropriate selection and duration of antimicrobial therapy.^90^ Surgical outcomes for patients with vertebral osteomyelitis are highly variable, with one‐quarter reporting residual pain and a similar proportion requiring repeat procedures.^91^ Anterior approaches of debridement and strut grafting with delayed posterior fusions with instrumentation may be an effective strategy in the context of vertebral osteomyelitis with associated spinal instability.^92^ In general, the paravertebral abscess can be managed by aspiration under CT guidance while epidural abscesses with associated neurological deficits should be managed with open drainage, bone debridement, and interbody fusion with or without fusion.^93^, ^94^

While Feldenzer et al established a reliable model of spinal epidural abscess formation, studies investigating the treatment of such abscesses in animal models have not been investigated.^43^ Further, few studies investigated the surgical management of spine related infection in an animal model. Chen et al describe a one‐stage surgical debridement of established pyogenic spondylodiscitis in dogs with autologous bone grafting followed by instrumentation and perioperative antibiotic therapy.^60^ Follow‐up radiological and macroscopic assessment showed no signs of recurrent infection after surgical debridement and treatment, although positive‐bacterial culture was observed in some cases despite no clinically relevant infection.^60^ The lack of studies on this topic highlights the need for relevant animal models of spine infection with indicated surgical management.

### 4.3 Designing a randomized control trial

Small animal models, such as mouse and rat models, use a more significant number of animals to offer statistical power and genetic diversity. The spine of the rat, in particular, is sufficient in size to be used in studies that require drilling and fixation techniques using implants. Access to the spine is easily achieved, especially in models of isolated discitis where the tail may be used for inoculation. However, small animals are limited by small bone diameters and incapable of supporting clinically relevant implant devices. Furthermore, as discussed previously, IV access is challenging to investigate the efficacy of prophylactic antibiotics.

Large animal models represent the human skeleton with higher fidelity than smaller animals, making them more relevant for the study of spinal infections. Dogs and sheep have been investigated for vertebral osteomyelitis, owing to their close anatomical dimensions with the human spine. These models allow for the use of approved clinical instrumentation to determine risks of postoperative infection without the need for implant scaling. Larger animals better tolerate multiple procedures than small animals and allow for easy IV access for antibiotic administration. Of course, considerations around ethics, cost per animal and housing over long time courses make large animal models more challenging to investigate. A canine model offers a unique opportunity to design and investigate antimicrobial treatments in randomized control trials through recruitment of canine patients presenting to veterinary clinics with ongoing infection. A large retrospective study of discospondylitis reported 513 dogs with an active infection that were treated with administration of antimicrobial drugs.^74^ Therefore, veterinary clinics offer an abundant population of patients, presenting with the natural disease that is suitable for randomized trials. This cohort of patients should be investigated as an intermediary step between a controlled, artificially induced preclinical model and a human clinical trial for promising interventional therapies targeting spinal infections.

## 5 CONCLUSION AND FUTURE PERSPECTIVES

While animal models for osteomyelitis have been given more attention in recent years, a spine specific model is crucial to replicate disease in the spine and the unique processes associated with it, addressing the unique anatomy of the spine, the avascular nature of its structures and tissues and the consequences of tissue destruction such as spinal cord compression. It is clear that hematogenously induced, and instrumentation‐associated spinal infection should be differentiated by the distinction in inducing processes, requiring different models. Systemic infection and localized discitis models may be useful to recapitulate hematogenous infection, while inoculated instrumentation models may be useful to study postoperative infection. Small‐animal models are not suitable for large instrumentation, and difficult IV access thwart antibiotic administration. In contrast, large‐animal models can be implanted with clinically relevant instrumentation and are resilient to repeat procedures. Long‐term prospective studies are necessary to determine the efficacy of treatment strategies, and therefore, continuous monitoring must be accessible to track progression. Further investigation is necessary to elucidate the specific mechanisms of host‐microbe response to inform antimicrobial therapy and administration techniques in a technically demanding body cavity. Use of advanced imaging techniques that incorporate bioluminescent bacterial strains is useful to track active infection. The development of new biomarkers, coupled with bioluminescent imaging and micro‐computed tomography, may provide even more precise and reliable data in vivo, elucidate molecular mechanisms and increase the impact of the study while ensuring that the clinically relevant question is adequately addressed. Veterinary clinics are a valuable source of relevant animal patients to investigate emerging technologies in this field.

## CONFLICT OF INTEREST

The authors declare no conflict of interest.

## AUTHOR CONTRIBUTIONS

Kieran Joyce and Daisuke Sakai contributed equally to the concept of the paper and writing of the article. Abhay Pandit provided substantial contributions to review format, revising the article critically and final approval. All Authors have read and approved the final submitted article.
